# First report from a single center retrospective study in Kazakhstan on acute myeloid leukemia treatment outcomes

**DOI:** 10.1038/s41598-021-03559-3

**Published:** 2021-12-14

**Authors:** G. U. Kulkayeva, V. M. Kemaykin, A. M. Kuttymuratov, Z. I. Burlaka, J. Z. Saparbay, G. T. Zhakhina, A. A. Adusheva, S. D. Dosayeva

**Affiliations:** 1 LLP «National Research Oncology Center», Kerey and Zhanibek Khandar Street 3, 01-0000 Nur-Sultan, Kazakhstan; 2 Department of Oncohematology and Stem Cell Transplantation, LLP «National Research Oncology Center», Kerey and Zhanibek Khandar Street 3, 01-0000 Nur-Sultan, Kazakhstan; 3 Department of Hepatology, Gastroenterology and Organ Transplantation, LLP «National Research Oncology Center», Kerey and Zhanibek Khandar Street 3, 01-0000 Nur-Sultan, Kazakhstan; 4 Department of Science, LLP «National Research Oncology Center», Kerey and Zhanibek Khandar Street 3, 01-0000 Nur-Sultan, Kazakhstan

**Keywords:** Cancer, Medical research

## Abstract

Acute myeloid leukemia (AML) is the most common hematological malignancy in adults. In the last decade, internationally approved AML treatment guidelines, including hematopoietic stem cell transplantation are widely used in Kazakhstan. The categorization of acute myeloid leukemia was done according to the French-American British classification. The prognosis of patients at the time of diagnosis was determined by cytogenetic tests following the guidelines of the European LeukemiaNet. The overall survival and event-free survival were analyzed using the Kaplan–Meier method, and hazard ratios were defined with Cox regression. In total, 398 patients with AML were treated in the National Research Oncology Center between 2010 and 2020. The mean age was 38.3 years. We found a correlation between ethnicity, cytogenetic group, white blood cell count, and treatment approaches with overall and event-free survival. There was a significantly longer OS in a cytogenetic group with a good prognosis compared with intermediate and poor prognosis. The median survival time in the group with a good prognosis was 43 months, 23 months in the intermediate group (p = 0.7), and 12 months in the poor prognosis group (p = 0.016). There was a significantly longer OS for the group of patients who received hematopoietic stem cell transplantation (HSCT), 52 months versus 10 months in the group who received chemotherapy only, p-value < 0.0001. Prognostic factors, such as cytogenetic group, initial WBC count, and treatment approaches are significantly associated with patient survival. Our study data were consistent with the most recent studies, available in the literature adjusted for the population in question.

## Introduction

According to the Kazakhstan National Cancer Institute, hematological malignancies represent 4.7% of all cancers, 5th place among both sexes in Kazakhstan. Morbidity due to hematological cancer is in 4th place among all cancer-related deaths in Kazakhstan^1^. Based on to Globocan data, 759 new cases of leukemia were registered in Kazakhstan in 2020, which corresponds to the 14th place among all cancers, the growth rate comprised 2% compared with 2019^2^.

Acute myeloid leukemia (AML) is the most common acute leukemia in adults^3^. There are no data regarding the estimated number of death due to AML in Kazakhstan. AML is a group of blood cell cancers originating from hematopoietic precursors in the bone marrow and resulting in a conglomeration of poorly differentiated myeloid cells infiltrating bone marrow, peripheral blood, and other organs^4^.

Blood disorder develops due to the acquisition of chromosomal translocations and multiple genetic mutations by leukemic stem cells^5–7^. Genetic events are usually associated with environmental influence, other blood disorders, tobacco use, and previous chemotherapy^8,9^. It is well known that AML incidence increases with age. The median age of people being diagnosed is 65 years^10^. Various factors were proved to influence the AML incidence and clinical outcome. The incidence increases with age with approximately 2 and 20 cases per 100,000 population for those under and over 65 years, respectively^11^. The male/female ratio is approximately 5:3^12^.

Currently, AML is the most common indication for hematopoietic stem cell transplantation (HSCT)^13^. Kazakhstan is the only country in Central Asia, where HSCT is available for patients with AML. Therefore, there are little data regarding AML epidemiology, treatment approaches, and clinical outcomes in Central Asia. Russian Federation is the most geographically close country to Kazakhstan. Bondarenko et al. from a single center in Russian Federation reported that 10-year overall survival after HSCT was 50% among patients with AML^14^. Here we present a single-center experience of AML treatment outcome in Kazakhstan.

## Methods

The data were retrospectively analyzed, and all ethical principles of the Helsinki Declaration have been followed. The Ethical Committee of the National Research Oncology Center (permit number №11) approved the study.

### Acute myeloid leukemia classification

The further division of AML into its subtypes was done according to the French-American British (FAB) classification. It sorts AML from M0 to M7, depending on the type of cell where leukemia developed and the maturity of these cells^15^. Moreover, this categorization takes into account the symptoms that show the whole picture of the patient’s condition.

### Molecular genetic stratification of patients by risk groups ELN 2017

Depending on disease pathogenesis, European LeukemiaNet defined three genetic groups for the classification of AML: poor cytogenetic prognosis, intermediate prognosis, and favorable prognosis^16^. The categorization was based on genetic abnormalities correlated with clinical characteristics. The poor prognosis included translocations t(6;9), t(9;22), inversions inv(3), deletions, mutations of RUNX1, ASXL1, and TP53. As intermediate ones were categorized t(9;11), mutations of NPM1 and FLT3-ITDhight, and anomalies not classified within poor or favorable prognosis. Translocations t(8;21), t(16;16), inversion of chromosome 16, RUNX1-RUNX1T1, and some mutations of NPM1 were categorized as favorable prognosis^17–19^.

### Induction therapy

Chemotherapy is the main treatment of AML to reach remission and complete response. The induction therapy was chosen according to the clinical protocol of diagnostics and treatment of AML approved by the Ministry of Health of the Republic of Kazakhstan^20^. Common procedures include cytarabine for 7 days, which is followed by 3 days of anti-tumor antibiotics. Mostly used anti-tumor antibiotics were daunorubicin (DNR), idarubicin (IDA), and doxorubicin (Doxo). There were other antibiotics used in the frame of chemotherapy, and they were united in one category “others”.

There were different complications after chemotherapy: multiple organ failure syndrome, disseminated intravascular coagulation, anal fissures, sepsis, paraproctitis, pneumonia, Hand-Foot Syndrome, candidiasis, polysinusitis, and others. Most common were febrile neutropenia, sepsis, and aspergillosis.

### Indications for HSCT and conditioning regimens

The clear indication for the HSCT was the first remission in the patients who had poor and intermediate cytogenetic prognosis groups, or the second remission in the patients who had intermediate and favorable prognosis according to cytogenetic analysis.

Reduced-intensity conditioning (RIC) regimens were carried out before HSCT according to the Fludarabine + Busulfan scheme (Fludarabin 30 mg/m^2^/day D-7 to D-2; Busulfan 10 mg/kg D-4, D-3). To carry out allogeneic HSCT, bone marrow, peripheral HSCs, or their combination was used as a source. The average number of HSC-CD 34 cells was 4.5 mln/kg.

### Statistical analysis

All data were analyzed with statistical software STATA 14.0. For bivariate analysis, the outcome was taken as the dependent variable, and association with predictors was checked with t-tests and chi-square tests. Kaplan–Meier curves were used to show the overall survival (OS) and event-free survival (EFS) of patients. A Long-rank test was used to access the equality of survival functions, and Cox regression was applied to define the Hazard Ratios. The significance level was set at 0.05.

### Ethical approval

The Ethical Committee of the National Research Oncology Center (permit number №11) approved the study.

## Results

In total, 398 patients with AML were treated in the National Research Oncology Center between 2010 and 2020. The final study included 371 patients, 27 patients were excluded from the study, patients were excluded if they were lost from follow-up or a history of prior treatment. Patients were predominantly younger than 60 years old (95.1%). The mean age of the study group was 38.3 (± 13) years old. There was no significant difference in the gender of the patients. Clinical-demographical characteristics are shown in Table 1.

**Table 1:** Demographic and medical characteristic of patients (N = 371).

Characteristic	Mean (SD) or N (%)	Bivariate analysis		
		Alive n = 134 (36.1%)	Died n = 237 (63.9%)	p-value
Age	38.3 (13)	36.4 (12.1)	39.3 (13.3)	0.043
**Gender**				0.189
Female	177 (47.7)	70 (40)	107 (60)	
Male	194 (52.3)	64 (33)	130 (67)	
**Relapse**				0.050
Yes	69 (18.6)	18 (26.1)	51 (73.9)	
No	302 (81.4)	116 (38.4)	186 (61.6)	
**White blood cell count**				0.060
WBC < 30 × ${10}^{9}$/L	240 (65)	95 (40)	145 (60)	
WBC $\ge 30 \times {10}^{9}$/L	131 (35)	39 (30)	90 (70)	
**AML classification (FAB)**				0.010
M0	52 (16)	8 (15.4)	44 (84.6)	
M0–M1	3 (0.9)	1 (33.3)	2 (66.7)	
M1	38 (11.7)	12 (31.6)	26 (68.4)	
M1–M2	76 (23.5)	24 (31.6)	52 (68.4)	
M2	50 (15.4)	21 (42)	29 (58)	
M3	24 (7.4)	15 (62.5)	9 (37.5)	
M4	44 (13.6)	21 (47.7)	23 (52.3)	
M4–M5	19 (5.9)	6 (31.6)	13 (68.4)	
M5	14 (4.3)	4 (28.6)	10 (71.4)	
M6	3 (0.9)	0	3 (100)	
M7	1 (0.3)	1 (100)	0	
**Cytogenetic prognosis**				0.027
Poor	31 (16.3)	6 (19.4)	25 (80.7)	
Intermediate	114 (60)	48 (42.1)	66 (57.9)	
Favorable	45 (23.7)	22 (49)	23 (51)	
**Type of induction therapy**				0.015
DNR	229 (62)	85 (37.1)	144 (62.9)	
IDA	50 (13.6)	26 (52)	24 (48)	
Doxo	42 (11.4)	13 (31)	29 (69)	
Other	48 (13)	10 (20.8)	38 (79.2)	
**Complications after chemotherapy**				
Febrile neutropenia				0.069
Yes	101 (27.2)	44 (43.6)	57 (56.4)	
No	270 (72.8)	90 (33.3)	180 (66.7)	
Sepsis				0.060
Yes	93 (25.1)	26 (28)	67 (72)	
No	278 (74.9)	108 (39)	170 (61)	
Aspergillosis				0.146
Yes	70 (18.9)	20 (28.6)	50 (71.4)	
No	301 (81.1)	114 (37.9)	187 (62.1)	
**Hematopoietic stem cell transplantation**				< 0.001
Yes	104 (28)	56 (54.4)	47 (45.6)	
No	267 (72)	78 (29.3)	188 (70.7)	

**Figure 1:**
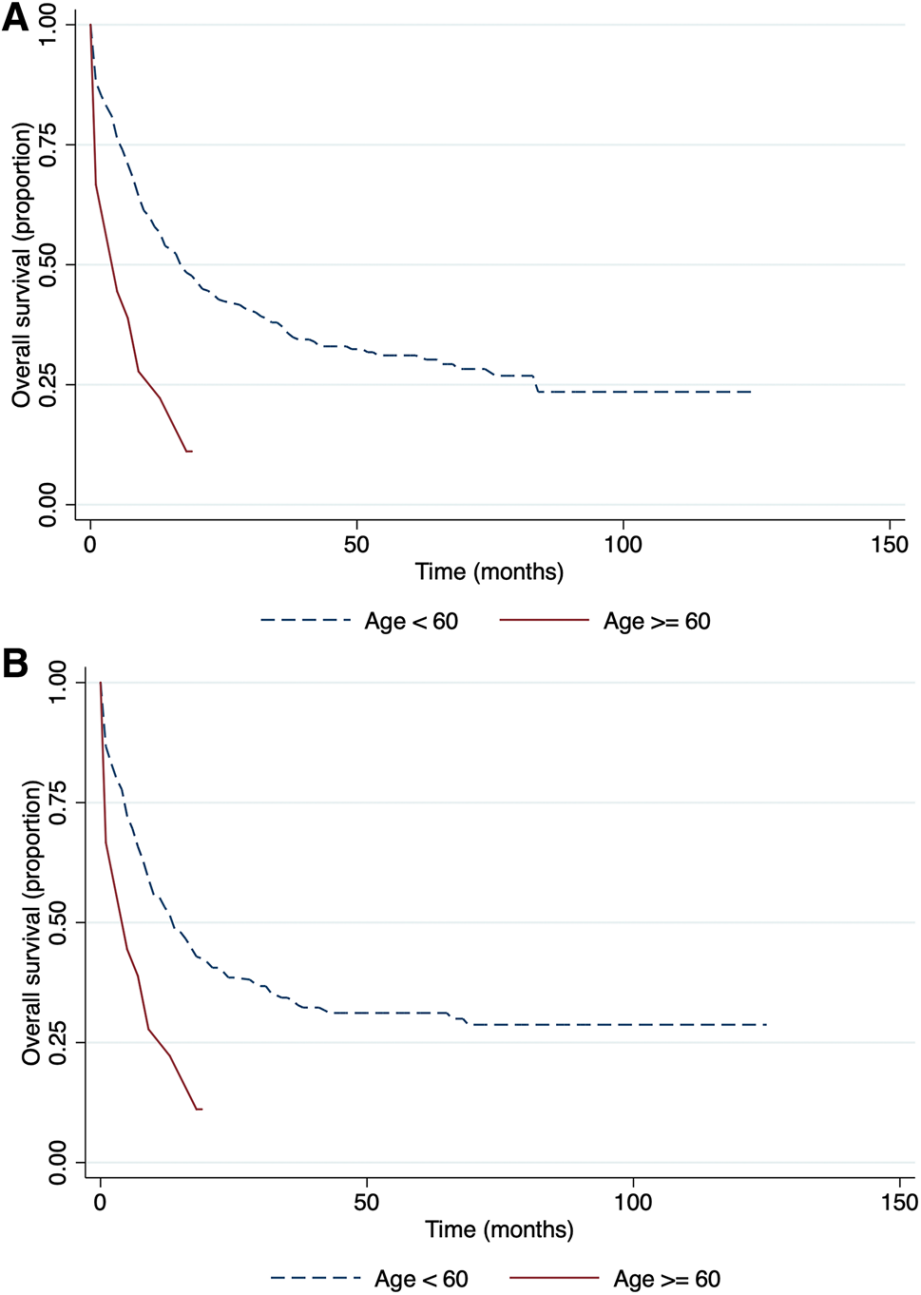
Age-related survival. (**A**) Overall survival and (**B**) event-free survival.

There was a statistically significant difference in overall survival (OS) and event-free survival (EFS) related to the patient's age. Median OS (Fig. 1A) of the 17 months for patients younger than 60 years old versus 4 months for the group of patients older than 60 years (p-value 0.0001; HR = 2.6). The median EFS (Fig. 1B) was 14 months for the younger group versus 7 months for the group older than 60 years (p-value 0.0010; HR-2.24).

**Figure 2:**
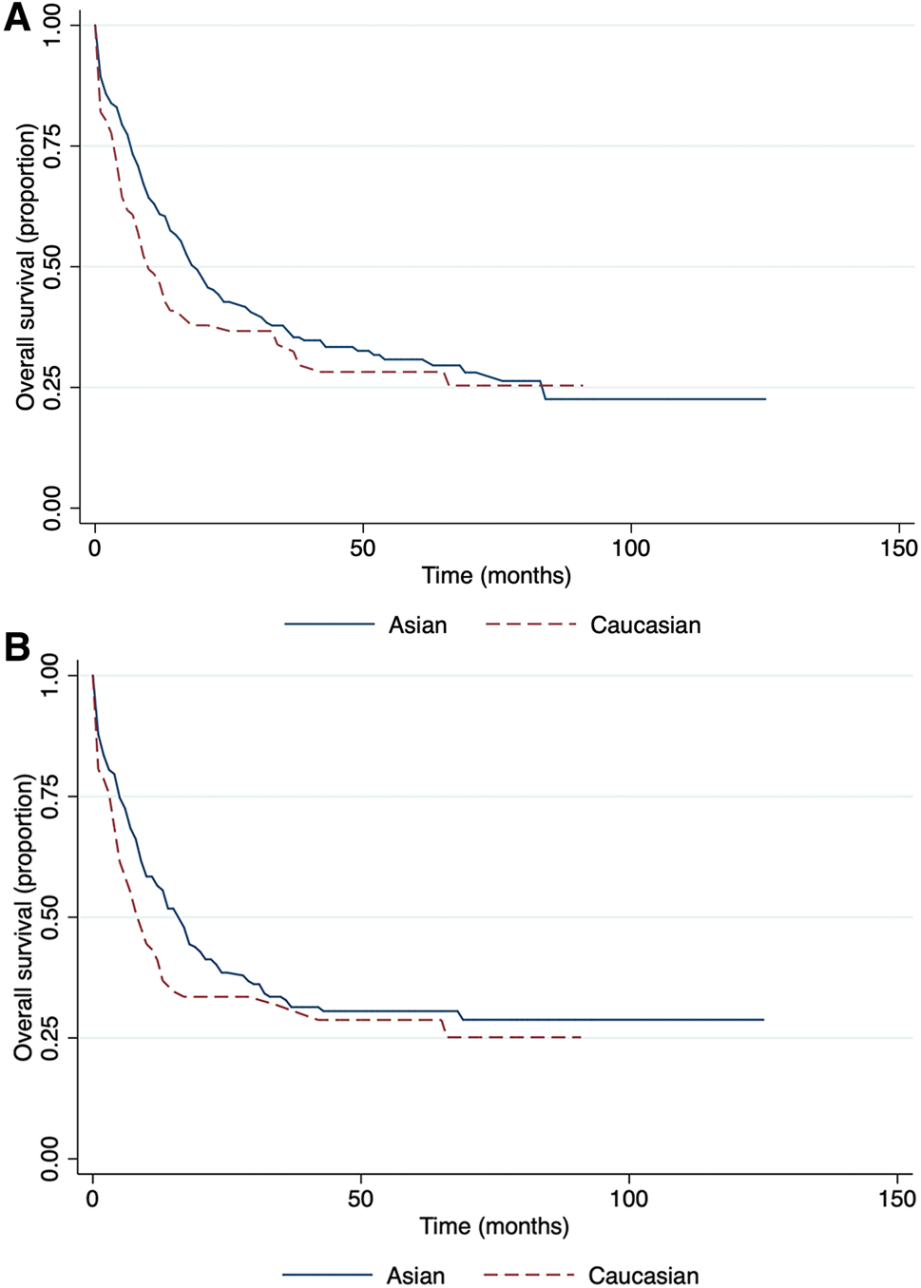
Race-related survival. (**A**) Overall survival and (**B**) event-free survival.

**Figure 3:**
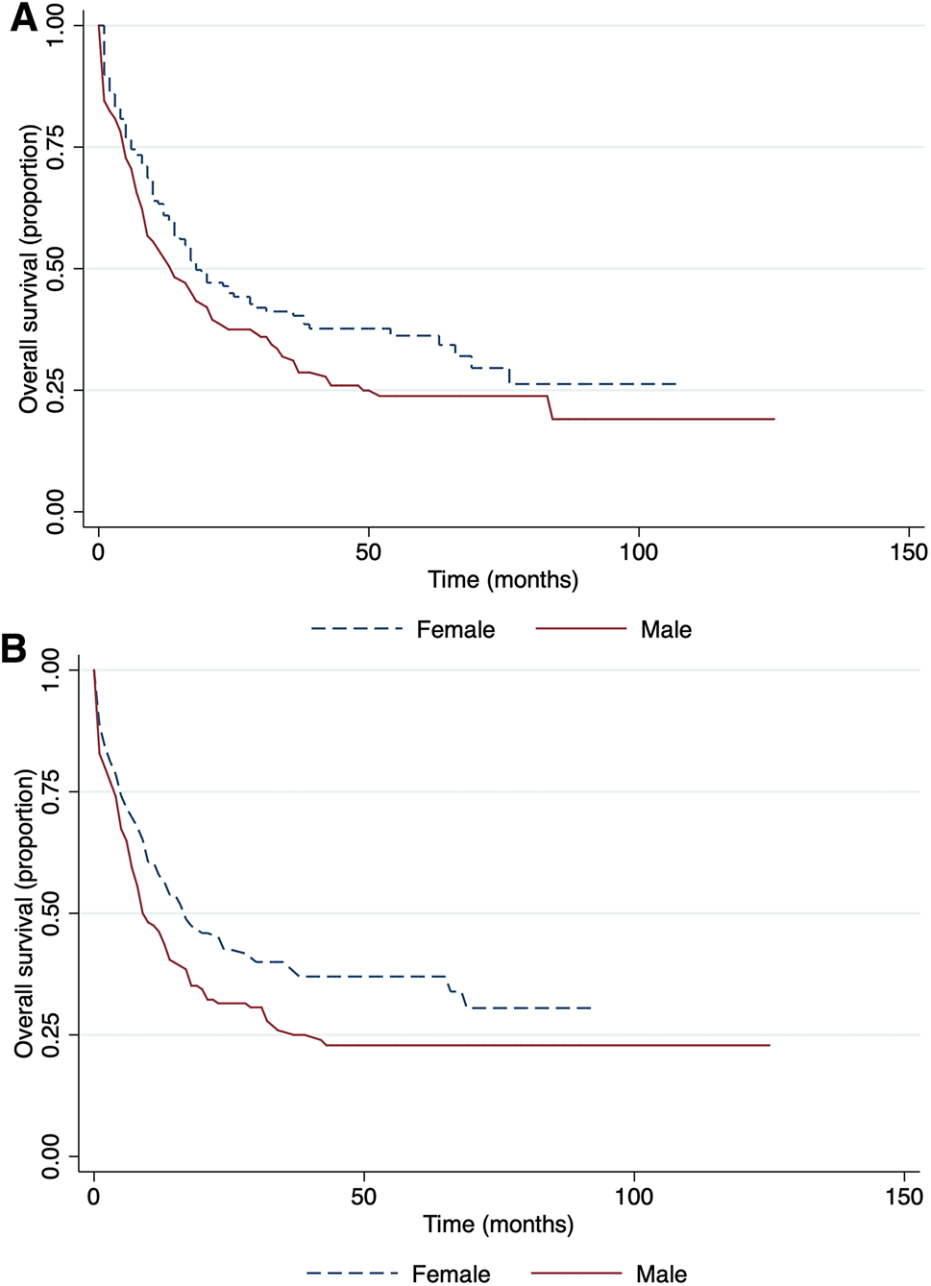
Gender-related survival. (**A**) Overall survival and (**B**) event-free survival.

Kazakhstan is a multinational country, in our cohort study we also compared OS and EFS related to race (Fig. 2). Our data showed that there was a significant difference in OS between the Asian and Caucasians group of patients, 19 and 10 months respectively (p = 0.0001).Our data showed that gender had no significance on the overall survival (Fig. 3A). The event-free survival was significantly related to gender (Fig. 3B). The median EFS in females was 18 months and 14 months in males.

**Figure 4:**
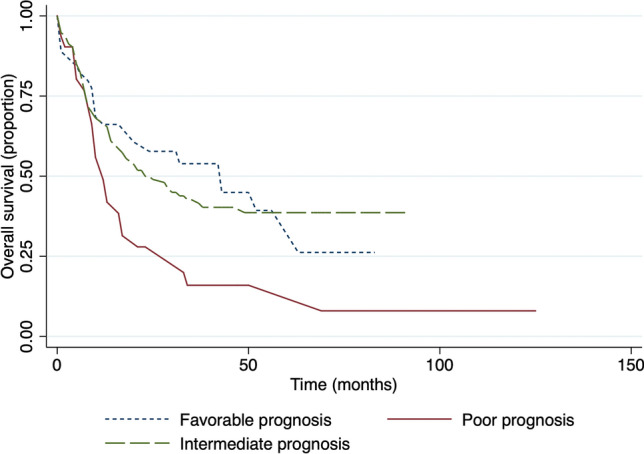
Overall survival in respect to cytogenetic prognosis.

**Figure 5:**
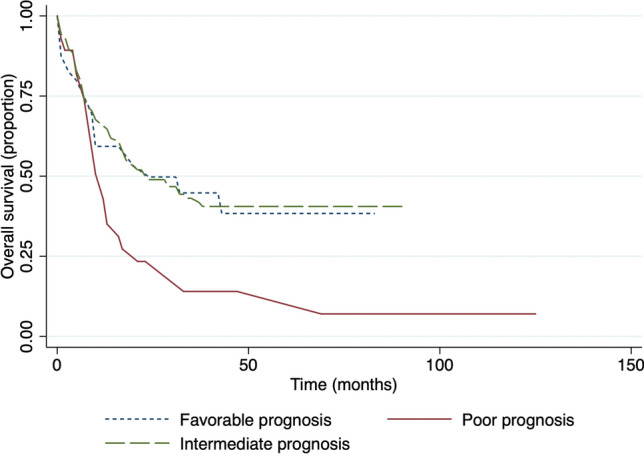
Event-free survival in respect to cytogenetic prognosis.

There was a significantly longer OS in a cytogenetic group with a good prognosis compared with intermediate and poor prognosis (Fig. 4). The median survival time in the group with a good prognosis was 43 months, 23 months in the intermediate group (p = 0.7), and 12 months in the poor prognosis group (p = 0.016). A similar significance was observed for EFS (Fig. 5), median survival in the favorable prognosis group was 24 months versus 11 months in the poor prognosis group (p = 0.036).

**Figure 6:**
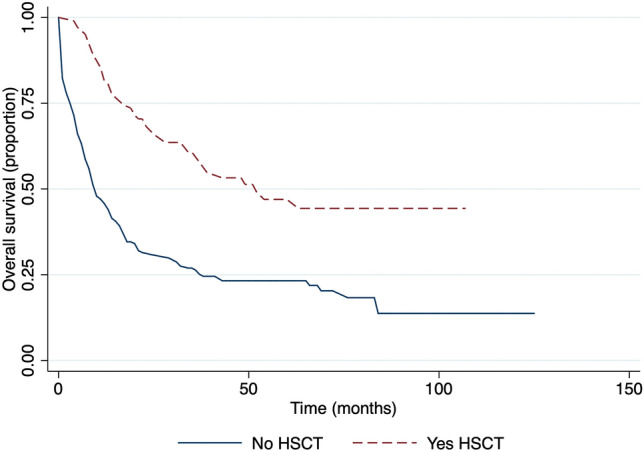
Survival in respect to hematopoietic stem cell transplantation.

There was a significantly longer OS for the group of patients who received hematopoietic stem cell transplantation (HSCT), 52 months versus 10 months in the group who received chemotherapy only, p-value < 0.0001 (Fig. 6).

**Figure 7:**
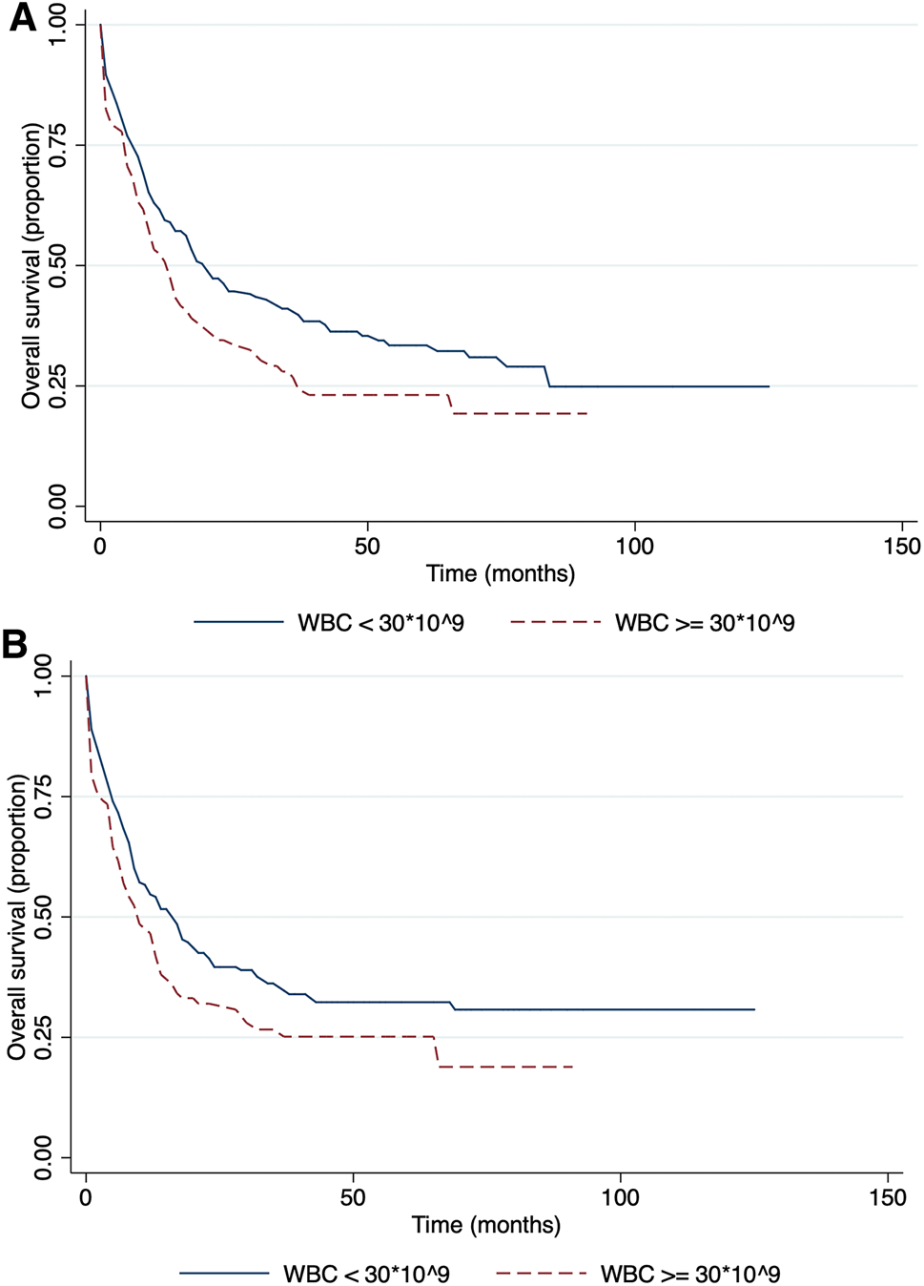
Survival in respect to white blood cell count. (**A**) Overall survival and (**B**) event-free survival.

**Figure 8:**
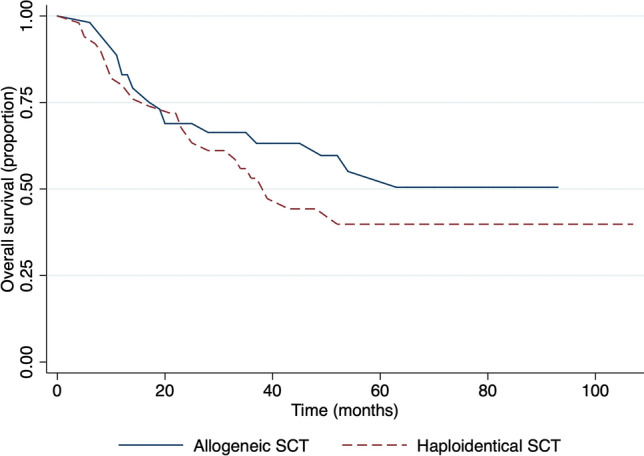
Overall survival in respect to the type of stem cell transplantation: allogeneic vs haploidentical.

Our analysis showed that OS and EFS in a group of patients with white blood cell (WBC) lower than 30 × 10^9^/L was significantly higher compared with the group with a WBC count of more than 30 × 10^9^/L at the time of diagnosis, 20 months vs 13 months and 17 months vs 10 months respectively (Fig. 7). The median survival time for those, who underwent allogeneic stem cell transplantation was 43 months, while for haploidentical stem cell transplantation patients it was 39 months, and the difference was not statistically significant, p = 0.28 (Fig. 8).

We found that the development of febrile neutropenia after chemotherapy was significantly associated with a negative outcome. In this study, 63.9% of patients died; 67% died due to the disease progression, the second most common cause of death was an infection.

## Discussion

The current study reports a 10-year single-center experience of AML treatment in Kazakhstan. The National Research Oncology Center is one of the largest centers in Central Asia. In our study, we analyzed the incidence, death, and other epidemiological data of AML, based on the medical record database of our center during the past 10 years. There is scarce epidemiological data on AML in Kazakhstan.

Although population data says that acute myeloid leukemia occurs mostly in adults^21^, the mean age of our patients was 38 years. It can be explained by the fact that initially there was a selection bias to perform hematopoietic stem cell transplantation. AML can be cured at younger ages more successfully than for those older than 60 years^22^. There is no clear cut-off point in literature for age to predict prognosis; however, the separation as younger than 60 and older showed significant results in both overall survival and event-free survival. Older individuals had for 160% more risk of death due to disease (Fig. 1A), and this result is consistent with other research outcomes^23^.

The data showed significantly better event-free survival for females (HR = 1.38). The trend of higher survival rates among women diagnosed with AML also was previously reported^24^. However, the cause of this phenomenon is still to be discovered. Moreover, according to our findings, ethnicity was also significantly associated with outcome, where Asians showed approximately two times longer median overall survival time compared to Caucasians. The impact of ethnicity on survival matches with the results of other countries, and such tendency could be explained with different genetic alterations among races^25^.

Similar to the literature, cytogenetic prognosis and white blood cell count were significantly related to the outcome. Both of these factors are key points in the diagnosis and treatment of AML. Consistent with the literature, the data of the present study shows that poor cytogenetic prognosis according to ELN classification at the time of diagnosis leads to higher mortality rates compared to intermediate and favorable ones^26^. For WBC count, a level at 30 × 10^9^/L was taken as a cut-off point and showed a significant association between high white blood cell count at the diagnosis time and lower overall and event-free survival of patients. It is consistent with the results of other researchers^27,28^. The possible explanation is that a high WBC count is associated with the possible progression of leukocytosis, which affects the prognosis of patients^29^.

The primary induction therapy with IDA showed a significant association with a favorable outcome compared to daunorubicin (DNR) and doxorubicin (Doxo), 52% vs 37% and 31% respectively. Hanyu Wang and colleagues performed a meta-analysis comparing the effect of different therapies on AML patient survival^30^. This could be a turning point to change the Kazakhstani protocol of AML treatment, where DNR and IDA are equally suggested for induction therapy.

Although the survival depending on the type of stem cell transplantation did not give statistical significance, it has medical importance for doctors and patients. The lack of donors for haploidentical stem cell transplantation is a big issue in Kazakhstan. Moreover, it should be noticed that the HSCT was initiated in our country only in 2010. This study gives an overview of the first 10-year experience’s results, implementation of acute myeloid leukemia treatment guidelines in Kazakhstan. The main limitation is a bias by age: the data is scarce of adults older than 60 years. It can be improved in further research, which can include all ages and be a prospective study.

## Conclusion

There is no doubt that allogeneic HSCT is one of the most effective methods for patients with AML. Significant progress in AML treatment has been achieved with the introduction of intensive chemotherapy with subsequent allogeneic HSCT. Until 2010 HSCT was not available in Kazakhstan. Our study revealed that HSCT for patients with AML is a preferable treatment method in comparison with chemotherapy alone.

This study has several limitations. First, it is a retrospective cohort study, with a small sample size. The lost to follow-up patients or missed data could be an issue. Further research with a prospective design of study could be done. In order to increase the generalizability of the findings, the data from several organizations that carry out the HSCT could be combined for analysis.

## Data Availability

The data that support the findings of this study are available from the corresponding author upon reasonable request.

## Abbreviations

AML: Acute myeloid leukemia
DNR: Daunorubicin
Doxo: Doxorubicin
EFS: Event-free survival
FAB: French-American British classification
HSCT: Hematopoietic stem cell transplantation
IDA: Idarubicin
OS: Overall survival
WBC: White blood cell
